# Moxibustion for primary dysmenorrhea: Protocol for a systematic review of randomized controlled trials

**DOI:** 10.1097/MD.0000000000018547

**Published:** 2020-01-03

**Authors:** Xiao Wu, Lu Gan, Yong Zhang, Bailu Chen, Jing Luo, Jue Yan, Guiquan Chen

**Affiliations:** Acupuncture Department, Hospital (T.C.M) Affiliated to Southwest Medical University, Luzhou, Sichuan, China.

**Keywords:** moxibustion, primary dysmenorrhoea, protocol, systematic review

## Abstract

**Background:**

Primary dysmenorrhea (PD) is one of the most common gynecological complaint among menstruating females. Acupuncture has been employed to relieve the pain-based symptoms and to avoid the side effects of conventional medication, especially, moxibustion has confirmed as an effective, convenient, and safe treatment for various types of menstrual pain. The purpose of this study is to systematically assess the effect and safety of moxibustion for treating PD.

**Methods and analysis:**

The following databases will be searched from their inception to December 2019: PubMed, Cochrane Central Register of Controlled Trials, MEDLINE, EMBASE, Wan-Fang Databases, China National Knowledge Infrastructure, Chinese Biomedical Literature Database, Citation Information by National Institute of Informatics, Chinese Scientific Journal Database. Two reviewers will search these databases, select data and evaluate the quality of studies separately. The methodological quality will be measured by the Cochrane risk of bias tool. The primary outcome is the pain degree evaluation including visual analog scale, numerical visual scale, verbal rating scale, Cox retrospective symptom scale, or any other scale used to evaluate the level of pain. And the response rate involved overall reduction in symptoms. The adverse effects, quality of life will be assessed as secondary outcomes. Risk ratio for dichotomous data and mean differences with a 95% confidence interval for continuous data will be adopted to express the effect and safety of moxibustion for PD.

**Trial registration number:**

PROSPERO CRD42019130141.

**Key Points**

(1) This systematic review will provide a systematic, objective, and comprehensive assessment of the effectiveness and safety of moxibustion for young females with primary dysmenorrhea.
(2) This review will provide new and helpful information for doctors, patients, and policymakers.
(3) The literature searching, trial screening and data extraction will be carried out independently by 2 authors.
(4) The languages only involved Chinese and English during electronic search will ignore the articles in Japanese, Korean, or other words.
(5) The various kinds of moxibustion and different stages may increase the risk of heterogeneity.

## 1 Introduction

Primary dysmenorrhea (PD) is one of the most common gynecological complaint that refers to menstrual pain in the lower abdominal region without an identifiable pathological condition among menstruating women, especially in adolescent and young females.^[1]^ According to different standards, the incidence of PD ranges from 45% to 95%, particularly 75% in reproductive females.^[2]^ Ten percent patients suffer from severe symptoms resulting in incapacity last 1 to 3 days each menstrual cycle.^[3]^ In different degree, PD leads to pain complain, activity limitation, efficiency impairment, and even life quality decrease.^[4,5]^ It is reported that school and work absenteeism caused by severe menstrual pain has been up to 14%.^[6]^

Previous studies have revealed that PD is attributed to high serum levels of prostaglandin E2, prostaglandin F2-aerfa, and leukotriene.^[7]^ Higher cytokine serum levels lead to severer pain and other associated symptoms.^[8]^ Currently, pharmacological therapies for PD mainly involved nonsteroid anti-inflammatory drugs (NSAIDs), which are considered as the first-line treatment for PD according to evidence-based medicine.^[9]^ Although NSAIDs and other drugs can alleviate the pain of PD significantly and quickly, their application is limited by gastrointestinal discomfort, fatigue, insomnia, and other complications, mostly because of their disability in curing the disease ultimately, along with their side effects and unaffordable high prices.^[10]^ Otherwise, It is reported that NSAIDs are associated with many adverse effects including indigestion, headache, drowsiness, and in 20% to 25% of patients, failure to relieve the pain was reported.^[11,12]^ Consequently, many females turn to seek complementary and alternative medicine to manage their menstrual complains including Chinese herbal medicine, acupuncture, and moxibustion which are identify effective, low-risk interventions. Acupuncture, as an indispensable component of traditional Chinese medicine (TCM), has been widely used in clinical treatment for PD all over the world.^[13,14]^ Particularly, moxibustion is not only effective and safe, but also very convenient for patients can treat with moxibustion by themselves at home.

Previous systematic reviews have displayed acupuncture is effective and safe and may have some advantages over routine medication treatment. In addition, moxibustion is able to relieve menstrual pain and other discomfort of PD patients in various pass ways, and there are lots of verifying study results to confirm the effects and safety of moxibustion for PD.^[15–17]^ However, no consensus has been reached on its efficacy, and it seems not that acknowledgeable by modern medicine currently. While there is still no critically designed systematic review to assess the effectiveness and safety of moxibustion for PD. Therefore, we decide to conduct a systematic review of moxibustion for PD to collect some reliable evidence for clinical guidance and to assistant PD females to seek more reasonable treatments. In this review, we aim to perform a systematic review to evaluate all the clinical studies on the effects and safety of moxibustion for treating PD.

## 2 Objective

The aim of this study is to systematically evaluate the effect and safety of moxibustion for treating PD, and provide high-quality evidence-based recommendations on the further treatment for doctors, patients, policy decision makers.

## 3 Methods

### 3.1 Study registration

This protocol for the systematic review has been registered on PROSPERO (registration number: CRD42019130141). The review reporting will be conducted in compliance with the preferred reporting items for systematic reviews and meta-analyses statement guidelines.

### 3.2 Study design

#### 3.2.1 Type of studies

All prospective randomized controlled clinical trials (RCTs) and quasi-RCTs will be included.

### 3.3 Type of participants

Patients suffered from PD will be included without course, ethnicity, disease duration, or disease severity restrictions.

### 3.4 Type of interventions

Any type of moxibustion therapy such as direct moxibustion, indirect moxibustion, heat-sensitive moxibustion, warm needling, natural moxibustion, moxa-burner moxibustion, crude drug moxibustion as the sole treatment or a part of combination therapy with other intervention will be included. Studies in which moxibustion is not used as a major intervention will be excluded.

### 3.5 Type of outcome measures

#### 3.5.1 Primary outcomes

(1) Pain: any other scale used to evaluate the level of pain including visual analog scale, numerical visual scale, verbal rating scale, Cox retrospective symptom scale.
(2) Clinic response rate: overall reduction in symptoms. As there are most Chinese clinic trials report outcomes based on a categorical assessment such as “significantly improved,” “improved,” “slightly better,” and “no effect,” the clinic effect rates will be evaluated by 3 different methods result from variation in effectiveness evaluation creates variation in results:
  (1) The “no effect” category will be classified as nonresponder and other categories as responder.
  (2) Improvement in symptoms by >50%will be classified as responder, while an improvement of <50% as nonresponder.
  (3) The categories “no change” and “worsening of symptoms” will be classified as nonresponder, while the category “shows improvement” as responder.^[18]^

#### 3.5.2 Secondary outcomes

(1) Adverse effects.
(2) Quality of life as measured by validated questionnaires.

### 3.6 Search methods

#### 3.6.1 Searching databases

##### 3.6.1.1 Electronic searches

A literature search will be conducted by LG, BC, and JL in the databases of PubMed, Cochrane Central Register of Controlled Trials, MEDLINE, EMBASE, Wan-Fang Databases, China National Knowledge Infrastructure, Chinese Biomedical Literature Database, Citation Information by National Institute of Informatics, Chinese Scientific Journal Database from their inception to December 2019.

### 3.7 Searching other sources

PROSPERO, the International Clinical Trials Registry Platform, and Clinical Trials.gov will also be searched to identify systematic reviews or ongoing/completed clinical trials. In addition, other resources will be searched manually such as the references of all included studies of this systematic review and general review. Theses and bibliographic references of included trials will also be reviewed.

### 3.8 Search strategy

The search strategy for electronic databases will adopted following items. The search term will be composed of the disease term part (eg, dysmenorrhea OR menstrual pain OR painful menstruation OR period pain OR painful period OR cramps OR menstrual disorder OR pelvic pain) and the intervention term part (eg, moxibustion OR moxa OR warm needling OR vesiculation OR blister).

Specific search strategies (eg, for PubMed) are as follows:

#1. ((((moxibustion [Title/Abstract]) OR moxa [Title/Abstract]) OR warm needling [Title/Abstract]) OR vesiculation[Title/Abstract]) OR blister [Title/Abstract]
#2. ((((((((dysmenorrhea [Title/Abstract]) OR PD [Title/Abstract]) OR menstrual pain [Title/Abstract]) OR painful menstruation [Title/Abstract]) OR period pain [Title/Abstract]) OR painful period [Title/Abstract]) OR cramps [Title/Abstract]) OR menstrual disorder [Title/Abstract]) OR pelvic pain [Title/Abstract])OR menstruation disturbances [Title/Abstract]
#3. (((random [Text Word] OR randomized [Text Word]) OR control [Text Word]) OR controlled [Text Word]) OR trial [Text Word] AND “humans” [MeSH Terms]
#4. #1AND#2AND#3

We will use similar search strategies for other electronic databases.

### 3.9 Study selection

**Figure 1:**
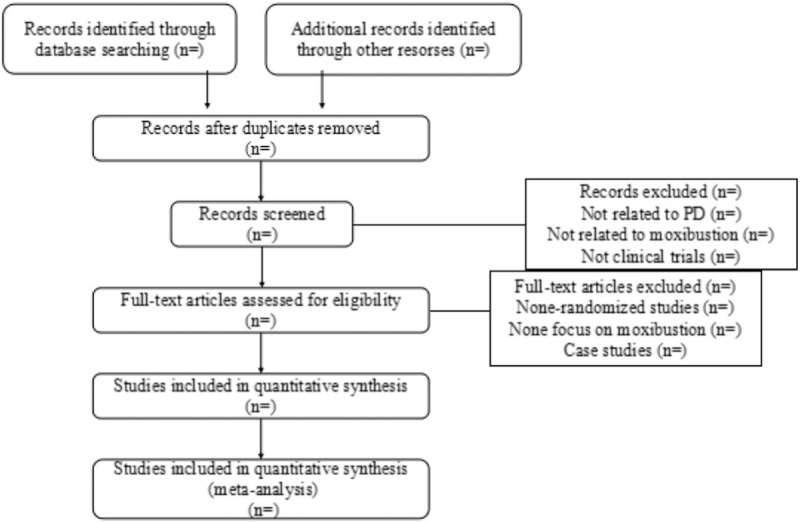
The PRISMA flow chart. PRISMA = preferred reporting items for systematic review and meta-analysis.

The data screening and selection process will be performed independently by LG and BC, then will be verified by XW. When disagreements on the selection are not resolved through team discussion, XW will make the decision. The details of review will be presented in the Figure 1.

### 3.10 Data extraction

Before beginning extraction, a consistency assessment will be performed through a pilot test, in which each of them will evaluate 2 trials, respectively. After making a common consensus, we will use a predefined extraction form to collect data from included trials, involving general information (name and year of publication, date of extraction, title of study, and author's publication details), study characteristics, eligibility criteria, interventions, outcome measurements, duration, adverse events, results, and type of moxibustion. For literature published in abstract or dissertation form with important details regarding methods and results missing, we will try to retrieve information from the authors.

### 3.11 Quality assessment

The Cochrane risk of bias tool, which is recommended by the Cochrane Reviewer's Handbook 5.0.24, will be used to evaluate the quality of the included studies. LG and BC will independently evaluate the quality of selected articles from the following 5 aspects: selection bias (random sequence generation or allocation concealment), performance bias and detection bias (blinding), attrition bias (incomplete outcome data), reporting bias (selective outcome reporting) and other biases. If necessary, we will contact the corresponding author to clarify issues. The result of the consistency evaluation will be presented with Kappa statistics, Kappa value <0.75 will be considered the consistency have reached. Any disagreements will be resolved through discussion or consultation with XW.

### 3.12 Data synthesis

Depending on its characteristics (participants, interventions, comparisons, and outcomes or methodology) of collected data, Analysis will be different. If there is excessive data heterogeneity, qualitative analysis will be performed to summarize the study. When meta-analysis is feasible, quantitative analysis will be used to process data, and each type of moxibustion intervention will be evaluated separately.

The review manager (RevMan V.5.3) software for Windows will be employed to carry out all statistical analyses. Before data meta-analysis, heterogeneity will be tested with a standard *χ*2 test. For studies with high heterogeneity (*P* > .10, *I*^2^ ≤ 50%), the fixed-effect model will be employed. For dichotomous and continuous data, the relative risk and mean difference with 95% confidence intervals will be expressed for evaluations respectively. For studies with low heterogeneity (*P* ≤ .10), subgroup or sensitivity analysis will be conducted. Ultimately, the total effect will be measured by using the *Z* score with significance set at *P* ≤ .05. Funnel plots will be used to detect the possibility of publication bias.

### 3.13 Subgroup analysis

If the necessary data are available, subgroup analysis will be conducted according to different factors as follows:

(1) Control interventions, such as sham/placebo moxibustion, no treatment, other TCM treatment or non-TCM treatment.
(2) Type of moxibustion, such as direct moxibustion, indirect moxibustion, beat-sensitive moxibustion, moxa burner moxibustion, warm needling, crude drug moxibustion, or natural moxibustion.
(3) Treatment frequency, such as less than 3 times per week versus more than 3 times per week.
(4) Intervention time of moxibustion treatment.
(5) Duration or dosage of moxibustion.
(6) Duration or severity of PD.
(7) Syndrome differentiation according to TCM theory.

### 3.14 Sensitive analysis

Sensitive analysis will be conducted after excluding low quality articles to identify whether the conclusions are robust. The “risk of bias” tool will be employed to evaluate the methodological quality of studies. The lower quality articles that have more than 3 “risk of bias categories” graded will be excluded, then we will perform a second meta-analysis. The results of the 2 meta-analysis will be compared, analyzed and discussed.

### 3.15 Patient and public involvement

Even though the patients will not be involved in the design of this study, development of the research question, and outcome measures will be informed by patients’ priorities, experience, and preference as reported in the published clinical studies in this domain. The results of this review will provide patients with new information on the credibility of current nonpharmacological treatments for treating PD.

## 4 Discussion

Dysmenorrhea usually accompanied by nausea, fatigue, headache, irritability, dizziness, vomiting, and diarrhea.^[5]^ Dysmenorrhea is divided into PD and secondary dysmenorrhea. PD is a very common gynecological disease characterized by abdominal pain before or during menstruation in young females.^[2]^ Previous studies have reported moxibustion as an effective, safety, and convenient alternative treatment is quilt optimal for PD, and increasingly number of PD patients are recommended to receive moxibustion to manage their complains during menstruation.^[15–17]^ However, the quality of current evidence has not been determined. Consequently, we will carry out a systematic review to provide more verifying evidence for doctors and patients, even policy makers. And the systematic review will provide a detailed summary of the present evidence for the effects of moxibustion in treating PD.

Even though moxibustion is commonly used in PD, no systematic reviews or meta-analysis on the effect and safety of moxibustion on PD have been reported.^[19]^ This study aims to provide a summary of the current evidence for moxibustion on PD, not only the mathematical synthesis of existing RCT results but also to provide details refer to clinical trial protocol development and clinic treatment. Furthermore, we also look forward to find predicting factors of clinical practice response by subgroup analysis. And the findings will be helpful to doctors, patients, and other people related to female healthcare.

However, there may be some potential limitations of the review. For example, the languages only involved Chinese and English during electronic search will ignore the articles in Japanese, Korean, or other words. Otherwise, the various kinds of moxibustion and different stages may increase the risk of heterogeneity. Finally, difficulty in blinding measures during moxibustion may lead to bias.

## Author contributions

GC is the guarantor of the article. XW designed and wrote the protocol, LG, YZ and JL advised on the design and performed data collection and analysis, BC, JL and LG searched the literature, selected studies and assessed data quality. All authors read and approved the final manuscript.
